# Supplementary material for: MicroRNA-Enriched Exosomes from Different Sources of Mesenchymal Stem Cells Can Differentially Modulate Functions of Immune Cells and Neurogenesis
Source: Biomedicines. 2021 Dec 30;10(1):69. doi: 10.3390/biomedicines10010069 (PMC8772751; doi:10.3390/biomedicines10010069)
Supplement: Supplementary file 1 [file biomedicines-10-00069-s001.zip › biomedicines-1459112-supplementary.pdf]

Supplementary Materials

Table S1. Details of samples used in this study.

S No	Sample ID	Tissue Source	Age	Gender
1	BM1820	Bone Marrow	30 yrs	Male
2	BM1916		35 yrs	Female
3	BM1931		40 yrs	Female
4	BM1932		42 yrs	Male
5	AD1901	Adipose Tissue	25 yrs	Male
6	AD1905		20 yrs	Male
7	AD1908		32 yrs	Female
8	AD1914		28 yrs	Female
9	WJ75	Wharton's Jelly	30 yrs	Female
10	WJ78		25 yrs	Female
11	WJ1901		28 yrs	Female
12	WJ1905		40 yrs	Female

Table S2. List of miRNAs associated with different disease and cellular pathways. found in IPA analysis.

Category	*p*-Value	Molecules
Developmental Disorder	3.21 × 10^{-46}–5.86 × 10^{-4}	let-7,mir-10,mir-103,mir-127,mir-130,**mir-143,mir-146,**mir-148,mir-15,mir-154,mir-155,mir-199,mir-21,mir-214,mir-22,mir-221,mir-25,mir-28,mir-30,mir-31,mir-320,mir-34,mir-378,mir-379,mir-432,mir-654
Inflammatory Disease	4.27 × 10^{-40}–4.54 × 10^{-3}	let-7,mir-10,mir-103,mir-130,**mir-143,mir-146**,mir-148,mir-15,mir-154,mir-155,**mir-181**,mir-186,mir-196,mir-199,mir-21,mir-214,mir-22,mir-221,mir-224,mir-23,mir-25,mir-26,mir-27,mir-28,mir-29,mir-30,mir-31,mir-320,mir-322,mir-34,mir-378,mir-379,mir-423,**mir-486**,mir-654
Respiratory Disease	4.27 × 10^{-40}–1.4 × 10^{-3}	let-7,mir-10,mir-103,mir-130,**mir-143,mir-146,**mir-148,mir-15,mir-154,mir-155,**mir-181**,mir-186,mir-191,mir-196,mir-199,mir-21,mir-214,mir-22,mir-221,mir-224,mir-23,mir-25,mir-26,mir-27,mir-29,mir-30,mir-31,mir-320,mir-322,mir-34,**mir-378,**mir-379,mir-423,mir-432,**mir-486**
Neurological Disease	6.49 × 10^{-40}–6.88 × 10^{-3}	let-7,mir-10,mir-103,mir-130,**mir-143,miR-145,mir-146**,mir-148,mir-15,mir-154,mir-155,**mir-181**,mir-186,mir-196,mir-199,mir-21,mir-214,mir-22,mir-221,mir-23,mir-25,mir-26,mir-27,mir-28,mir-29,mir-30,mir-31,mir-320,mir-322,mir-34,mir-423,MIR4516
Cellular Growth and Proliferation	7.5 × 10^{-24}–6.05 × 10^{-3}	let-7,mir-10,mir-103,mir-130,**mir-143,mir-146**,mir-148,mir-15,mir-154,mir-155,**mir-181**,mir-186,mir-191,mir-196,mir-199,mir-21,mir-214,mir-22,mir-221,mir-224,mir-23,mir-25,mir-26,mir-27,mir-28,mir-29,mir-30,mir-31,mir-320,mir-322,mir-34,**mir-378**,mir-379,**mir-486**,mir-654
Cell Death and Survival	6.42 × 10^{-22}–6.73 × 10^{-3}	let-7,mir-10,mir-130,**mir-143,mir-146**,mir-148,mir-15,mir-154,mir-155,**mir-181**,mir-186,mir-191,mir-199,mir-21,mir-214,mir-22,mir-221,mir-23,mir-25,mir-26,mir-27,mir-29,mir-30,mir-320,mir-322,mir-34,**mir-378**,mir-379,**mir-486,**mir-493,mir-654
Inflammatory Response TH1 and TH2 activation pathway	5.79 × 10^{-18}–6.05 × 10^{-3}	let-7,mir-10,mir-130,**mir-143,mir-146**,mir-148,mir-15,mir-154,mir-155,**mir-181**,mir-199,mir-21,mir-22,mir-221,mir-23,mir-25,mir-27,mir-29,mir-30,mir-31,mir-320,mir-34,**mir-378**,mir-423,**mir-486**,mir-654

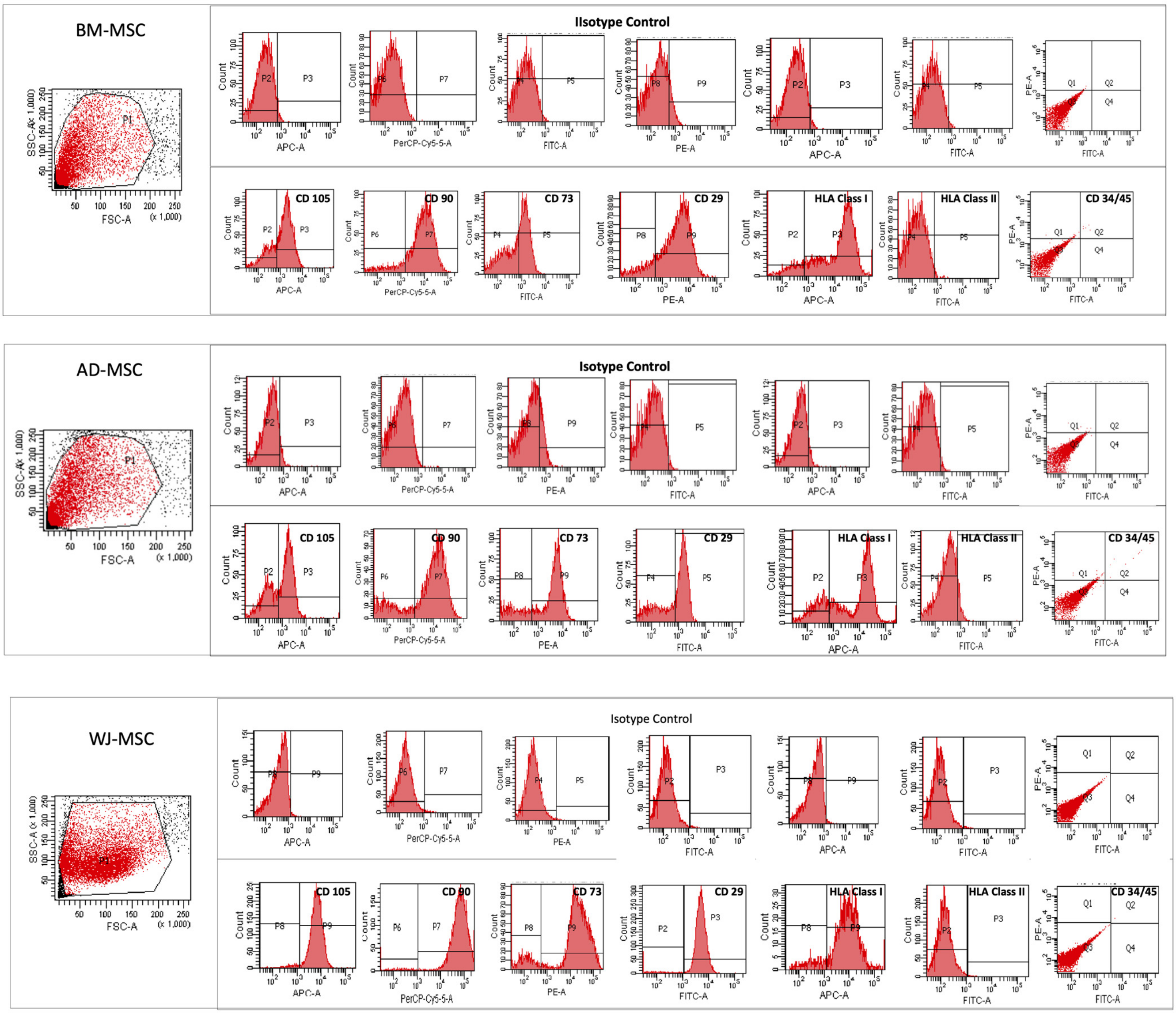

Figure S1. Flow cytometric analysis of hMSCs showing expression of positive surface markers CD90, CD 105, Cd29, CD73 and HLA Class-I and expression of negative surface markers HLA Class-II and CD34/45.

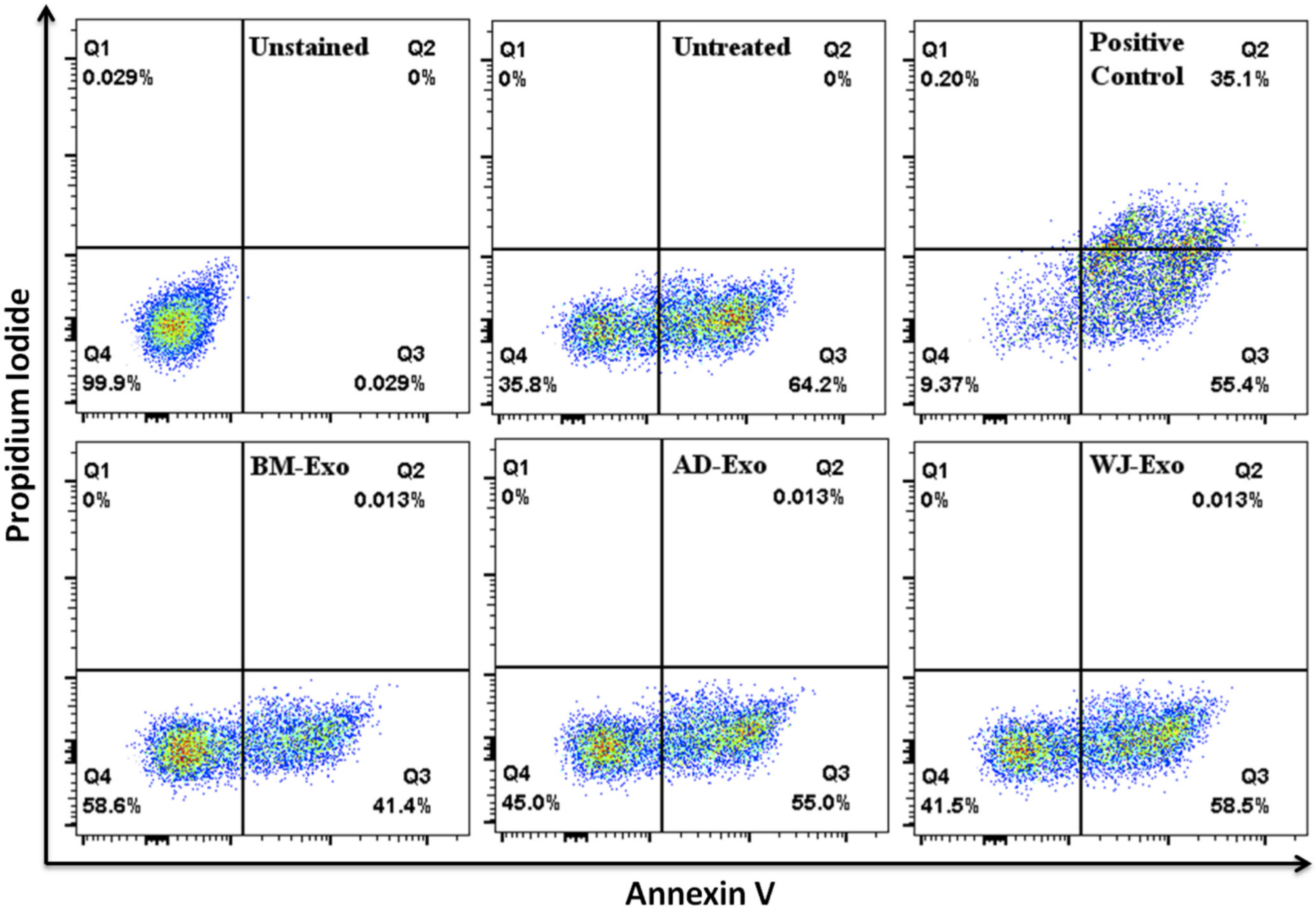

Figure S2. Two-dimensional dot plots representing early apoptotic cells upon treatment of neutrophils with MSCs-Exosomes. Annexin V/PI double staining kit has been used in flow cytofluorimetric analyses for detecting cellular apoptosis, while propidium iodide (PI) is used to detect necrotic or late apoptotic cells. The data generated by flow cytometry are plotted in two-dimensional dot plots in which PI is represented versus Annexin V-FITC. These plots can be divided in four regions corresponding to: (1) viable cells (PI/FITC −/−; Q4); (2) early apoptotic cells (PI/FITC −/+; Q3); (3) late apoptotic cells (PI/FITC +/+; Q2); (4) necrotic cells (PI/FITC +/−; Q1). .

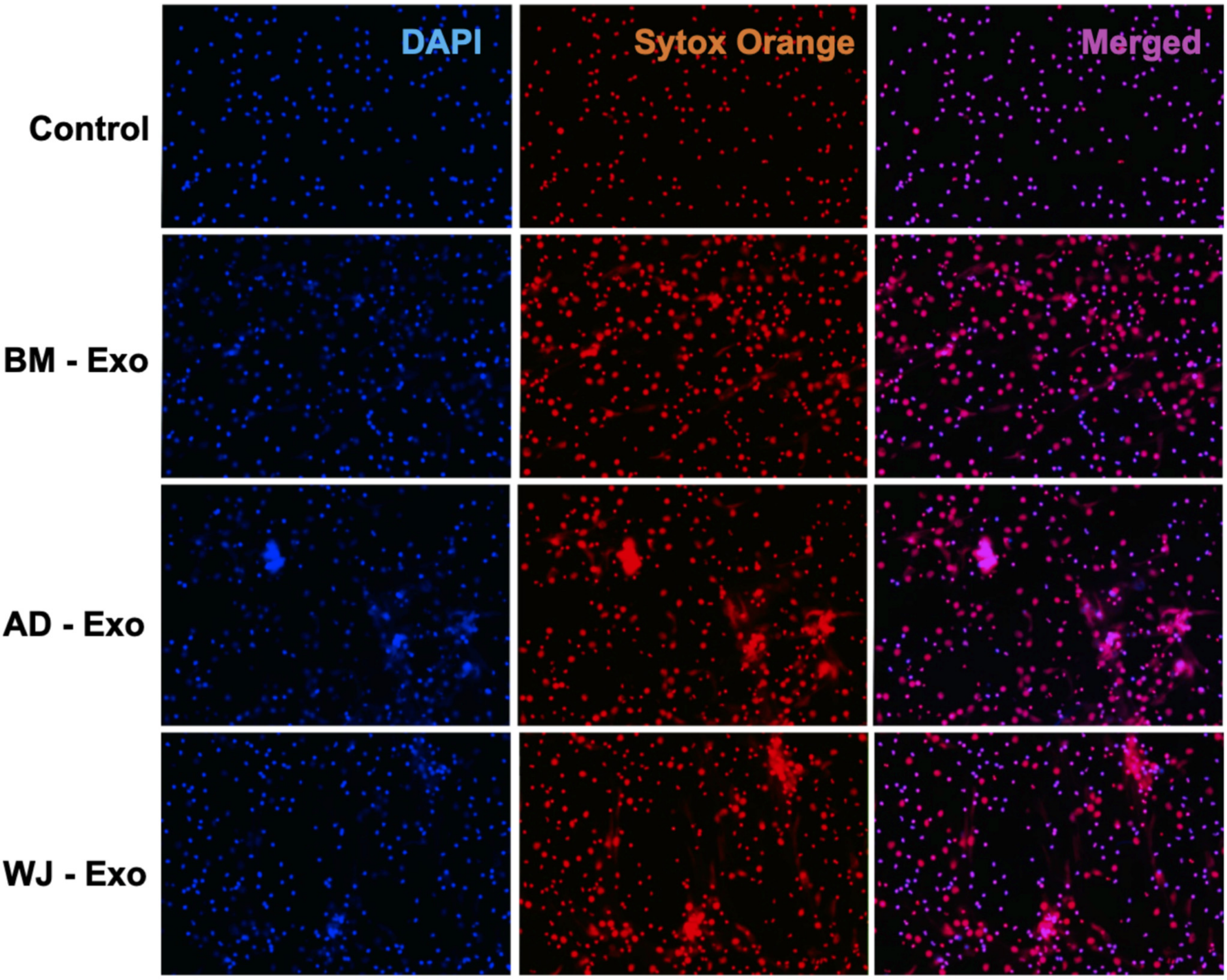

Figure S3 Representative images of neutrophils after 24 h post incubation in presence or absence of MSCs exosomes. Neutrophils were seeded in a 96 well clear bottom plate at a seeding density of 0.2 × 10^6 cells per ml and incubated with exosomes for 6 hours. After incubation the cells were fixed with PFA and stained with sytox orange (25 μM) and DAPI for 15 min. The cells were then observed in ImageXpress Micro Confocal High content imaging System (Molecular Devices).
